# Quantum teleportation in Heisenberg chain with magnetic-field gradient under intrinsic decoherence

**DOI:** 10.1038/s41598-024-60321-1

**Published:** 2024-04-26

**Authors:** Seyed Mohammad Hosseiny, Jamileh Seyed-Yazdi, Milad Norouzi, Patrizia Livreri

**Affiliations:** 1 Physics Department, Faculty of Science, Vali-e-Asr University of Rafsanjan, Rafsanjan, Iran; 2 Department of Engineering, University of Palermo, Palermo, 90128 Italy; 3 Department of Electronics, San Diego State University, San Diego, CA USA

**Keywords:** Quantum teleportation, Quantum estimation, Magnetic-field gradient, Intrinsic decoherence, Quantum physics, Quantum information

## Abstract

One of the most appealing quantum communication protocols is quantum teleportation, which involves sharing entanglement between the sender and receiver of the quantum state. We address the two-qubit quantum teleportation based on the Heisenberg XYZ chain with a magnetic-field gradient affected by intrinsic decoherence. An atomic spin chain is primarily coupled to the linear gradient of the magnetic field in the x-direction, with the assumption that the magnetic field varies linearly with the position of the atom. By using the concepts of fidelity and average fidelity in the presence of the magnetic field gradient and under the effect of intrinsic decoherence in the current model, and considering the variables of the system, an improved quantum teleportation can be achieved. In addition, using the concept of remote quantum estimation, we examine remote quantum sensing in this article, which is very useful in quantum communication.

## Introduction

Quantum teleportation is undeniably one of the most thrilling subjects in quantum communication^1–12^. In this process, which was proposed by Bennett et al.^13^, Alice (sender) transfers an unknown quantum state to Bob (receiver) by sharing a classical or non-classical channel^14–16^. This technique can be achieved over short distances or across thousands of kilometers using existing equipment^17^.

Initially, quantum teleportation was conducted based on photons^18^, and subsequently with diverse systems such as trapped ions^19,20^, atomic ensembles^21^, high-frequency phonons^22^, and several other systems^23–26^. Now, this popular protocol is acknowledged as a crucial technique for implementing numerous quantum protocols, including measurement-based quantum computing^27^, quantum repeaters^28,29^, and fault-tolerant quantum computing^30^.

Studying quantum teleportation in dense matter systems at finite temperatures can be fascinating. In Ref.^31^, the investigation of quantum teleportation, dense coding, and entanglement based on the XYZ spin chain model influenced by Kaplan Shekhtman Entin wohlman-Aharony (KSEWA) interaction and Dzyaloshinskii-Moriya (DM) interaction have been studied. Furthermore, quantum teleportation via thermal mixed states in XXX Heisenberg chain systems has been investigated^32^. Moreover, the impact of an external magnetic field on the standard teleportation protocol has been reported in the context of a two-qubit XY model^33^. Further, the discussion of thermal entanglement and teleportation in the XXZ chain with varying DM interactions has been addressed^34^. Besides, quantum Fisher information of an output state of the teleportation in the Heisenberg XYZ chain with magnetic Field and KSEWA Interaction at thermal equilibrium has been studied in X and Z directions^35^.

Intrinsic decoherence, which is usually included by the Milburn method^36^ in open quantum systems, denotes decoherence quite independent of any environment. This process resembles decoherence (i.e., one where phase coherence or phase interference is suppressed over time) while this process is intrinsic to nature, i.e., not determined by simply averaging over ’external’ degrees of freedom that happen to be entangled with the desired system^37^. Therefore, intrinsic decoherence differs from environmental decoherence which arises from the nature of the surrounding environment of open quantum systems and is ineluctable. Distinguishability between intrinsic and environmental decoherence in experiments and theory is reported in Ref.^37^. Quantum correlation dynamics influenced by intrinsic decoherence in a Heisenberg spin chain model under Dzyaloshinskii-Moriya interaction have been investigated^38^. Moreover, quantum memory-assisted entropic uncertainty relation in a Heisenberg spin chain model under intrinsic decoherence has been explored^39^. Further, the qutrit teleportation via XXZ Heisenberg chain affected by an inhomogeneous magnetic field under intrinsic decoherence has been examined^40^. As well as the robustness of an output state of quantum teleportation through a two-qubit Heisenberg chain model influenced by dipole interaction and magnetic field under intrinsic decoherence has been addressed^41^. The entanglement teleportation using an XYZ Heisenberg chain model affected by various interactions under intrinsic decoherence is also examined^42^. In addition, the examination of quantum teleportation via the entangled channel consisting of a two-qubit XYZ Heisenberg chain model influenced by DM interaction in the presence of intrinsic decoherence has been reported^43^. Also, the quantum teleportation and phase quantum estimation according to the two-qubit XYZ Heisenberg chain model influenced by dipole and symmetric cross-interactions influenced by intrinsic decoherence have been explored^44^. The quantum teleportation through the entangled states including a two-qubit XYZ Heisenberg chain model driven by a uniformly magnetic external field as a channel under intrinsic decoherence has been investigated^45^. Moreover, the exploration of using helical spin chains for quantum teleportation to share entanglement has been studied^46^. The study of qutrit teleportation and entanglement involving the one-axis counter-twisting Hamiltonian under intrinsic decoherence has also been explored^47^.

Our motivation is investigating the two-qubit quantum teleportation based on the Heisenberg XYZ chain with magnetic-field gradient in the presence of intrinsic decoherence. Consider an atomic spin chain that is primarily coupled to the linear magnetic-field gradient in the x-direction, with the assumption that the magnetic field varies linearly with the atom’s position^48^. This makes our model distinguishable from other XYZ Heisenberg chain models and those done in the aforementioned references. Note that, the intrinsic decoherence is incorporated into the current model using the Milburn method^36^. Moreover, to delve deeper into quantum teleportation using the existing model, we aim to calculate the initial phase within the teleported state, potentially holding vital encoded information.

Additionally, to further investigate quantum teleportation based on the current model, we aim to estimate the initial phase in the teleported state, which may contain crucial encoded information or reveal the nature of the process that prepared the initial state^49^. In addition, since we cannot ignore the interaction between the system and its surrounding environment, we also examine the dynamics of open quantum systems^50–55^ in this article. According to this, the dynamics with respect to the flow of information in systems theory are divided into two classes: Markovian and non-Markovian. If information continually flows from the system to the environment, the dynamic is called Markovian. However, if information can be periodically returned to the system from the environment due to quantum memory effects, the dynamics in this class become non-Markovian^56–58^. In open quantum systems, quantum teleportation depends on the nature of system evolution.

The structure of our article consists of four parts: After the introduction, quantum communication preliminaries are defined in “The physical model” section. In “Results and discussion” section, we introduce the physical model that can serve as a resource for quantum teleportation. Finally, in “Conclusion” section our conclusions are given.

## Preliminaries

### Quantum teleportation

The standard protocol for remote transmission^59^ involves a two-qubit mixed state $\rho _{ch}$, serving as a channel or resource and represented by a generalized depolarized quantum channel $\Lambda (\rho _{ch})$ based on a single-qubit input state $\rho _{in}$. Alice’s goal is to send her encoded qubit to Bob using this method. The unknown input (initial) state of teleportation can be considered to be an arbitrary pure single-qubit state as follows:

1 $$\begin{aligned} |\psi _{in}\rangle =\text {cos}\Big (\frac{\theta }{2}\Big )|0\rangle +e^{i\phi } \text {sin}\Big (\frac{\theta }{2}\Big )|1\rangle , \end{aligned}$$

where $\theta$ and $\phi$ represent the amplitude and phase of the initial state of teleportation. The teleportation output state, in the teleportation of an arbitrary single-qubit state (input state $\rho _{in}=|\psi _{in}\rangle \langle \psi _{in}|$)), can be assumed by^59^:

2 $$\begin{aligned} \rho _{out}=\Lambda (\rho _{ch})\rho _{in}=\sum _{i=0}^{3}Tr [{\mathcal {B}}_{i}\rho _{ch}]\sigma _{i}\rho _{in}\sigma _{i}, \end{aligned}$$

in which $\Lambda (\rho _{ch})$ denotes a generalized depolarized channel and ${\mathcal {B}}_{i}$ represents the Bell state corresponding to the Pauli matrix $\sigma _{i}$ that is defined by:

3 $$\begin{aligned} {\mathcal {B}}_{i}=\Big (\sigma _{0}\otimes \sigma _{i}\Big ){\mathcal {B}}_{0} \Big (\sigma _{0}\otimes \sigma _{i}\Big ),\,\,\, i=1,2,3, \end{aligned}$$

that we have $\sigma _{0}={\mathbb {I}},\sigma _{1}=\sigma _{x},\sigma _{2} =\sigma _{y},\sigma _{3}=\sigma _{z}$ and ${\mathbb {I}}$ refers to the identity matrix. Furthermore, for any two arbitrary qubits, each defined in base $\{|0\rangle ,|1\rangle \}$, and we have ${\mathcal {B}}_{0}=\frac{1}{2}\Big (|00\rangle +|11\rangle \Big )\Big (\langle 00|+\langle 11|\Big )$ such that, the teleportation channel is initially prepared in the maximum entangled state of Bell states.

The degree of similarity between two quantum states and the quality of the teleported state is determined by the criterion of fidelity $f\Big (\rho _{in}(t),\rho _{out}(t)\Big )$, which is defined by^60,61^:

4 $$\begin{aligned} f\Big (\rho _{in}(t),\rho _{out}(t)\Big )=\Bigg (Tr \Big (\sqrt{\sqrt{\rho _{in}(t)} \rho _{out}(t)\sqrt{\rho _{in}(t)}}\Big )\Bigg )^2, \end{aligned}$$

As well as one can obtain the average fidelity of teleportation $f_{av}$ as follows:

5 $$\begin{aligned} f_{av}:=\frac{1}{4\pi }\int _{0}^{2\pi }d\phi \int _{0}^{\pi }f\Big (\rho _{in}(t), \rho _{out}(t)\Big )sin(\theta )d\theta . \end{aligned}$$

The threshold of the maximum classical average fidelity occurs at $f_{av}=2/3$; afterward, we go into the quantum average fidelity. The closer the quantum average fidelity is to the unit, represents that optimum quantum teleportation can occur.

Here, we assume Alice transmits two encoded qubits to Bob. For an arbitrary two-qubit pure state, the unknown input (initial) state can be considered as:

6 $$\begin{aligned} |\psi _{in}\rangle =\text {cos}\Big (\frac{\theta }{2}\Big )|10\rangle +e^{i\phi } \text {sin}\Big (\frac{\theta }{2}\Big )|01\rangle , \end{aligned}$$

To determine the output state $\rho _{out}$ of an arbitrary two-qubit state, one can generalize Eq. (2) as^62^:

7 $$\begin{aligned} \rho _{out}=\sum _{i,j=0}^{3}p_{ij}\Big (\sigma _{i}\otimes \sigma _{j}\Big ) \rho _{in}\Big (\sigma _{i}\otimes \sigma _{j}\Big ). \end{aligned}$$

in which $\sum p_{ij}=1$ and $p_{ij}=Tr [{\mathcal {B}}_{i}\rho _{ch}]Tr [{\mathcal {B}}_{j}\rho _{ch}]$.

### Quantum phase estimation

#### Quantum Fisher information

The accuracy of phase estimation is crucial, and it is determined by the “Cramér-Rao (CR) inequality”. This inequality compares the difference between the true and estimated phase values, representing the estimation accuracy, with the “quantum Fisher information (QFI)^63–66^” that defines the lower limit of accuracy based on the number of measurement repetitions. Therefore, the QFI is a powerful tool for estimating the uncertain true value of a phase. The quantum CR inequality^66,67^ can be expressed as follows:

8 $$\begin{aligned} \Delta \phi \ge \frac{1}{\sqrt{{\mathcal {F}}_\phi }}, \end{aligned}$$

which gives us the smallest detectable phase change phase $\phi$. Also, ${\mathcal {F}}_\phi$ represents the QFI with respect to $\phi$ and is defined by^63,68^:

9 $$\begin{aligned} {\mathcal {F}}_\phi =\sum _{i}\frac{(\partial _{\phi }\lambda _{i})^2}{\lambda _{i}}+4\sum _{i\ne j}\frac{(\lambda _{i}-\lambda _{j})^2}{\lambda _{i}+\lambda _{j}}|\langle \varphi _{i}| \partial _{\phi }\varphi _{j}\rangle |^2. \end{aligned}$$

such that $|\varphi _{i}\rangle$ and $\lambda _{i}$ are eigenvectors and eigenvalues of the density matrix, respectively. According to the theory of quantum estimation, increasing the QFI means improving the estimation accuracy. We use this point in quantum phase estimation to improve remote quantum sensing. The question is, what is the mechanism of remote quantum sensing in this article? In response, it can be said that the quantum estimation process is conducted by sensors, and their proper design can significantly enhance measurement accuracy. For instance, a quantum sensor can be based on a qubit that encodes information in the relative phase of its quantum state by interacting with a weak external field. The information obtained by measuring the qubit can be used to estimate the electric field, magnetic field, or temperature^69–73^. In many cases, it may not be feasible to be physically present for a special assessment due to security risks or logistical constraints. However, remote estimation offers a solution, allowing individuals to realize quantum remote sensing using classical and quantum communication channels, even when the necessary tools are not physically available at the desired location. In this work, we investigate the idea of remote parameter estimation, which so-called remote quantum sensing, using two-qubit quantum teleportation in the presence of magnetic field gradient and intrinsic decoherence. More precisely, in Alice’s location, there is a qubit whose desired information is encoded in its initial state phase, and Alice is obliged to teleport the state of this unknown qubit to Bob, who is equipped with a sensitive sensor for estimation. In addition, Bob is under the effect of magnetic field gradient and intrinsic decoherence such that their effects on the quality of remote estimation, teleportation process, and their optimization are investigated.

## The physical model

Consider a paradigmatic open quantum system consisting of a two-qubit anisotropic Heisenberg XYZ chain in the presence of external magnetic fields. The system’s Hamiltonian can be given by^74,75^:

10 $$\begin{aligned} \begin{aligned} {\mathcal {H}}= J_x (\sigma _x \otimes \sigma _x)+J_y (\sigma _y \otimes \sigma _y)+J_z (\sigma _z \otimes \sigma _z)+(\omega _j+b) \sigma _z \otimes I+(\omega _j-b) I \otimes \sigma _z, \end{aligned} \end{aligned}$$

where $\sigma _i (i = x, y, z)$ denote the Pauli matrices, $J_i (i = x, y, z)$ represent the interaction coefficients in the ferromagnetic $J_i>0$ and anti-ferromagnetic $J_i<0$ couplings between two spin degrees of freedom, respectively. It should be noted that the difference between $J_x$ and $J_y$ measures the anisotropy of the system. Moreover, we consider the external magnetic fields to be along the z-direction. $\omega _j$ is transition frequency dependent on the magnitude of the magnetic field $B_1$, while *b* measures the degree of the non-uniformity of the field, and *I* is the identity matrix.

The eigenstates $\left| \Phi _j\right\rangle (j=1,2,3,4)$ and the corresponding eigenvalues $E_j$ of the Hamiltonian 10 on standard basis $\{|11\rangle ,|10\rangle ,|01\rangle ,|00\rangle \}$, can be expressed by

11 $$\begin{aligned} \begin{aligned}{}&\left| \Phi _1\right\rangle =\eta _{+}|11\rangle +\epsilon _{+}|00\rangle ,\,\,\,\,\,\,\,\,\, E_1=J_z+\delta , \\&\left| \Phi _2\right\rangle =\eta _{-}|11\rangle +\epsilon _{-}|00\rangle ,\,\,\,\,\,\,\,\,\, E_2=J_z-\delta , \\&\left| \Phi _3\right\rangle =\nu _{+}|10\rangle +\xi _{+}|01\rangle ,\,\,\,\,\,\,\,\,\, E_3=\chi -J_z, \\&\left| \Phi _4\right\rangle =\nu _{-}|10\rangle +\xi _{-}|01\rangle ,\,\,\,\,\,\,\,\,\, E_4=-\chi -J_z, \end{aligned} \end{aligned}$$

where

$$\begin{aligned} \begin{aligned}{}&\eta _{ \pm }= \pm \frac{1}{\sqrt{2}} \frac{2 \omega _j \pm \delta }{\sqrt{\delta ^2 \pm 2 \omega _j \delta }} \\&\epsilon _{ \pm }= \pm \frac{1}{\sqrt{2}} \frac{J_x-J_y}{\sqrt{\delta ^2 \pm 2 \omega _j \delta }} \\&\nu _{ \pm }= \pm \frac{1}{\sqrt{2}} \frac{\chi \pm 2 b}{\sqrt{\chi ^2 \pm 2 b \chi }}, \\&\xi _{ \pm }=\frac{1}{\sqrt{2}} \frac{J_x+J_y}{\sqrt{\chi ^2 \pm 2 b \chi }}, \end{aligned} \end{aligned}$$

such that $\delta =\sqrt{4 \omega _j^2+\left( J_x-J_y\right) ^2}$ and $\chi =$ $\sqrt{4 b^2+\left( J_x+J_y\right) ^2}$. In Eq. (11) we used $$|0\rangle =\left( \begin{array}{c} 1 \\ 0 \\ \end{array}\right)$$ and $$|1\rangle =\left( \begin{array}{c} 0 \\ 1 \\ \end{array}\right)$$, such that $|ij\rangle$ denotes $|i\rangle \otimes |j\rangle$ in which $i,j=0,1$.

In Ref.^36^, Milburn suggested a straightforward modification of the Schrodinger equation, adding a term that accounts for the decay of quantum coherence in the energy eigenstate basis. This modification is based on the assumption that for sufficiently brief time intervals, the system does not continuously evolve under unitary transformation. Based on consideration of the effect of intrinsic decoherence, the time evolution of the density matrix of the system is given by the Milburn equation as follow:

12 $$\begin{aligned} \begin{aligned}{}&\rho (t)=\sum _{m, n} \exp \left[ -\frac{\gamma t}{2}\left( E_m-E_n\right) ^2-i\left( E_m-E_n\right) t\right] \times \left| \Phi _m\right\rangle \left\langle \Phi _m|\rho (0)| \Phi _n\right\rangle \left\langle \Phi _n\right| , \end{aligned} \end{aligned}$$

where $E_{m,n}$ and $|\Phi _{n,m}\rangle$ are, respectively, the eigenvalues and the eigenvectors of the Hamiltonian of the system Eq. (10), $\gamma$ is the intrinsic decoherence rate. Besides, $\rho (0)$ denotes initial density matrix.

Now, if we assume the qubits 1 and 2 are both initially in the spin-down states, i.e., the system is in an unentangled state $|\Psi (0)\rangle =|00\rangle$ at the beginning, then we have $\rho _0=|\Psi (0)\rangle \langle \Psi (0)|$. By a straightforward computation from Eqs. (11, 12), one can calculate the time evolution of the density matrix of the system on the standard basis as

13 $$\begin{aligned} \begin{aligned} \rho (t)=&\alpha |11\rangle \langle 11|+\mu | 11\rangle \langle 00|+\mu ^*|00\rangle \langle 11|+\beta | 00\rangle \langle 00|, \end{aligned} \end{aligned}$$

in which

$$\begin{aligned} \begin{aligned}{}&\alpha =1-\frac{1}{2 \delta ^2}\left( \delta ^2-4 \omega _j^2\right) \left[ 1-\cos (2 \delta t) \exp \left( -2 \delta ^2 \gamma t\right) \right] , \\&\mu =\frac{1}{\delta ^2}\left( J_x-J_y\right) \times \left\{ \omega _j-\frac{1}{2}[2 \omega _j \cos (2 \delta t)-\textrm{i} \delta \sin (2 \delta t)] \exp \left( -2 \delta ^2 \gamma t\right) \right\} , \\&\beta =\frac{1}{2 \delta ^2}\left( J_x-J_y\right) ^2\left[ 1-\cos (2 \delta t) \exp \left( -2 \delta ^2 \gamma t\right) \right] . \end{aligned} \end{aligned}$$

It is evident that the time-dependent density operator of the system is independent of the non-uniform magnetic field *b*.

**Figure 1:**
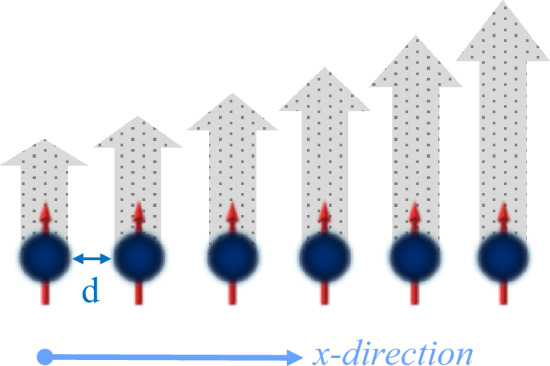
An atomic spin chain (Blue spheres) is coupled to a linear magnetic field gradient (Gray arrows) in the x-direction. Each atom is separated by a distance *d*.

At the moment, we assume an atom to have two hyperfine spin states. Hence, when an atom is coupled to a magnetic field, the energy splitting between two hyperfine states is changed because of the Zeeman effect^48,76^. Determining the difference in transition frequencies of two atoms at different locations can distinguish the magnetic-field gradient. As mentioned in^48^, an atomic spin chain can be employed to probe the magnetic-field gradient as illustrated in Fig. 1, where each atom is separated by a distance *d*. Consider that the magnetic field $B(x_j)$ linearly changes with the position $x_j$ as follows:

14 $$\begin{aligned} B(x_j)=B_1+Gx_j, \end{aligned}$$

in which $B_1$ represents the reference magnetic field and *G* denotes the magnetic-field gradient. The transition frequency $\omega _j$ depends on the magnitude of the magnetic field $B(x_j)$ and defined as

15 $$\begin{aligned} \omega _j=\omega _0+\lambda B(x_j). \end{aligned}$$

such that $\omega _0$ indicates the transition frequency of the two hyperfine spin states without the external magnetic field and $\lambda$ represents the gyromagnetic ratio of an atom.

It is important to note that the key aspect of this work, which distinguishes our work from other works, is here that by replacing the definitions (Eqs. 14 and 15) in the Hamiltonian (Eq. 10), we consider the magnetic field including the gradient varies with the position of atoms. In the following, we consider Eq. (13) as a resource for two-qubit quantum teleportation and analyze the results. Another point is that to plot the figures throughout the work, we employ the nondimensionalized parameter method as described in^44,77,78^.

## Results and discussion

First, we calculate the output state of the two-qubit quantum teleportation performed based on the computed resource in Eq. (13). By placing Eqs. (14 and 15) in Eq. (10) and using Eqs. (6 and 7), the output state of two-qubit quantum teleportation is obtained as follows:

16 $$\begin{aligned} \rho _{out}(t)=\left( \begin{array}{cccc} 0 &{} 0 &{} 0 &{} 0 \\ 0 &{} \rho _{22} &{} \rho _{23} &{} 0 \\ 0 &{} \rho _{32} &{} \rho _{33} &{} 0 \\ 0 &{} 0 &{} 0 &{} 0 \\ \end{array} \right) \end{aligned}$$

where the non-vanishing elements of the output density matrix are given by

17 $$\begin{aligned} \begin{aligned}{}&\rho _{22}=\sin ^2 \left( \frac{\theta }{2}\right) ,\,\,\,\,\,\,\,\rho _{22}=\cos ^2 \left( \frac{\theta }{2}\right) , \\&\rho _{23}=\frac{ 2 \sin (\theta ) (J_x-J_y)^2 (\omega _j)^2}{\delta ^4} \Bigg (\exp \Big (2 \gamma t \delta ^2\Big )-\cos \left( 2 t \delta \right) \Bigg )^2 \Big (\exp \left( -4 \gamma t \delta ^2+i \phi \right) \Big ),\\&\rho _{32}=\rho ^{*}_{23}. \end{aligned} \end{aligned}$$

where $\delta$ and $\omega _j$ are defined in Eqs. (11 and 15).

One of the most important criteria that should always be considered in quantum teleportation is the average fidelity, which determines the success rate of a quantum teleportation. Therefore, we examine the qualitative behavior of average fidelity in two-qubit teleportation according to the current model in this section.

**Figure 2:**
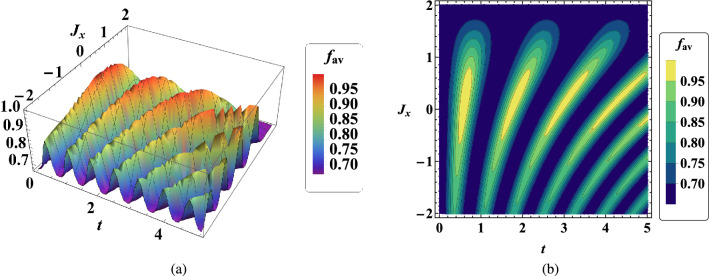
(**a**) Temporal variations of average fidelity $f_{av}$ in two-qubit teleportation in terms of $J_x$ when $B_1=0.5$, $\omega _0=0.9$, $G=1$, $x_j=1$, $J_y=2$ , $\gamma =\lambda =0.001$. (**b**) Contour plot of temporal variations of $f_{av}$ with the same conditions.

In Fig. 2a, time evolution of average fidelity $f_{av}$ in terms of interaction coefficient $J_x$ is plotted. The initial observation from this figure states that the average fidelity $f_{av}$ exceeds 2/3, suggesting an enhancement in quantum teleportation. Moreover, based on the prediction, we observe that in the initial times of quantum teleportation, there is a better quality of teleportation because the average fidelity is higher in the initial times and decreases over time. In addition, it can be seen that when the value of $J_x$ is around zero, we have a better average fidelity value. Of course, the value of the average fidelity is higher in the ferromagnetic region $J_x>0$ than in the antiferromagnetic region $J_x<0$. These results can also be obtained in Fig.  2b, which is the contour plot of this process. Note that the same results can be obtained for $J_y$.

**Figure 3:**
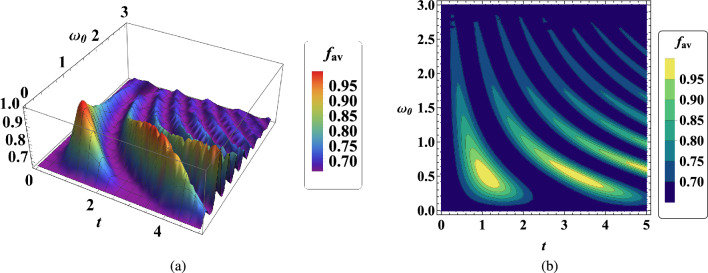
(**a**) Time evolution of average fidelity $f_{av}$ in two-qubit teleportation versus $\omega _0$ when $B_1=0.5$, $G=1$, $x_j=1$, $J_x=2$, $J_y=1$ , $\gamma =\lambda =0.001$. (**b**) Contour plot of time evolution of $f_{av}$ with the same conditions.

In the next step, the temporal behavior of the average fidelity $f_{av}$ in terms of transition frequency $\omega _0$ of the two hyperfine spin states without the external magnetic field is plotted in Fig. 3. Quantum teleportation has better quality at low transition frequencies, i.e. less than $\omega _0<1$. Furthermore, the qualitative behavior of average fidelity $f_{av}$ becomes more oscillate over time.

**Figure 4:**
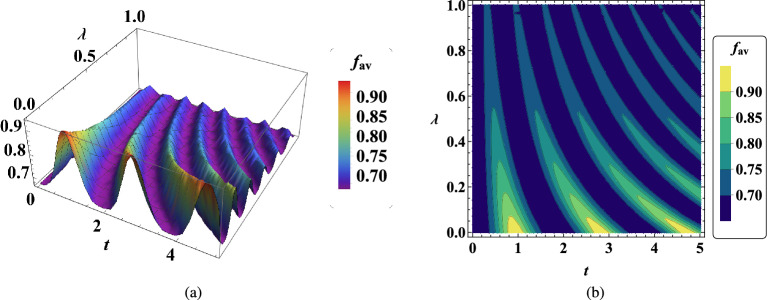
(**a**) Dynamic of average fidelity $f_{av}$ in two-qubit teleportation vs $\lambda$ when $B_1=0.85$, $\omega _0=0.7$ $G=0.9$, $x_j=1$, $J_x=2$, $J_y=1.1$, $\gamma =0.001$. (**b**) Contour plot of time evolution of $f_{av}$ with the same conditions.

In Fig. 4, the average fidelity dynamic is illustrated in terms of the gyromagnetic ratio $\lambda$ of an atom. The significant point is that the quantum teleportation in the present model is optimal when the gyromagnetic ratio of the atom is less than 0.2, therefore, the more this ratio is less than 0.2, the better the quality of quantum teleportation is achieved. The gyromagnetic ratio corresponds to the ratio of the magnetic momentum in a particle to its angular momentum^79^. Therefore, it can play a crucial role in practical quantum teleportation^80^.

**Figure 5:**
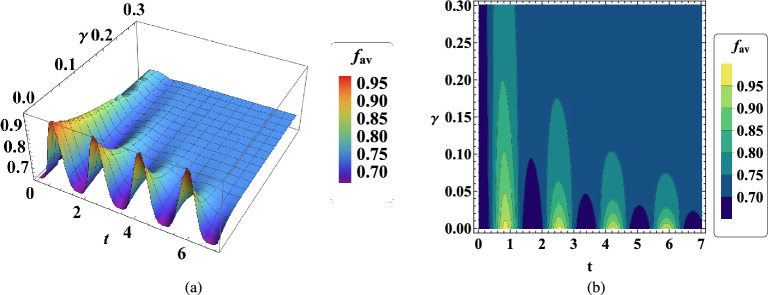
(**a**) The temporal behavior of average fidelity $f_{av}$ in two-qubit teleportation in terms of $\gamma$ when $B_1=0.65$, $\omega _0=0.75$ $G=1$, $x_j=1$, $J_x=2$, $J_y=0.9$, $\lambda =0.001$. (**b**) Contour plot of time evolution of $f_{av}$ with the same conditions.

In the next stage, the time evolution of the average fidelity $f_{av}$ in terms of the intrinsic decoherence rate $\gamma$ is investigated in Fig. 5. The average fidelity value clearly decreases as the intrinsic decoherence rate increases. This means that the system is affected by decoherence effects^81^, including noise, and causes quantum teleportation to be adversely affected. So increasing these effects can cause the teleportation process to fail.

**Figure 6:**
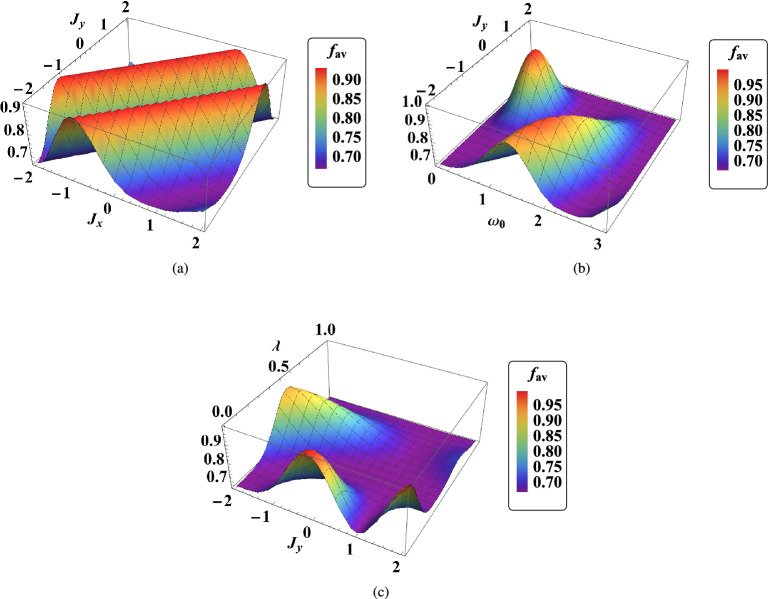
The qualitative behaviors of average fidelity $f_{av}$ in two-qubit teleportation in terms of (**a**) $J_y$ vs $J_x$ when $B_1=1$, $\omega _0=0.7$ $G=0.4$, $x_j=1$, $\gamma =\lambda =0.01$, (**b**) $J_y$ vs $\omega _0$ when $B_1=0.8$, $G=2$, $x_j=1$, $J_x=2$, $\gamma =\lambda =0.001$, and (**c**) $\lambda$ vs $J_y$ when $B_1=0.4$, $\omega _0=0.5$, $G=2$, $x_j=1$, $J_x=1$, $\gamma =0.004$. Here, we consider $t=1$.

In Fig. 6a, the temporal behavior of average fidelity in terms of interaction coefficients of $J_x$ and $J_y$ is depicted. It is evident that when both interaction coefficients $J_x$ and $J_y$ have the same value, the quality of quantum teleportation is reduced. This is because the value of average fidelity is minimum at the points where $J_x=J_y$. This is obvious because the direction of the magnetic field is only in the z-direction. Moreover, as we see in Eqs. (13 and 16) the density matrix and the output density matrix of quantum teleportation are independent of $J_z$. Note that, $J_i$ ($i=x,y$) measures the anisotropy of the system. When we have $J_x=J_y$, the model is reduced to the Heisenberg XXZ. Since our system is independent of $J_z$ and we have $J_x=J_y$, then our system becomes an isotropic system, and this causes a decrease in the value of the average fidelity and therefore decreases the quality of quantum teleportation. Hence, the quality of the two-qubit quantum teleportation with magnetic-field gradient when the model is reduced in the Heisenberg XXZ is suppressed. Besides, in Fig. 6b, the qualitative behavior of average fidelity $f_{av}$ versus $J_y$ and $\omega _0$ is shown. We see that when $J_y>0$ is in the ferromagnetic region, the maximum value of average fidelity occurs in $\omega _0<1$; But, when $J_y<0$ is in the anti-ferromagnetic, the maximum value of the $f_{av}$ arises in $1<\omega _0<2$. Moreover, in Fig. 6c, the qualitative behavior of average fidelity $f_{av}$ versus $J_y$ and $\lambda$ is illustrated. We see that when we have $\lambda =0.5$ and $J_y=0$ or 2, then the quantum teleportation can be optimum. It means that the role of the anisotropy in the system under magnetic-field gradient is very important. Furthermore, in these figures, it is obvious that the role of the ferromagnetic region is significant. Figure 6a–c refer to the optimal quantum teleportation that is dependent on the interaction coefficients.

**Figure 7:**
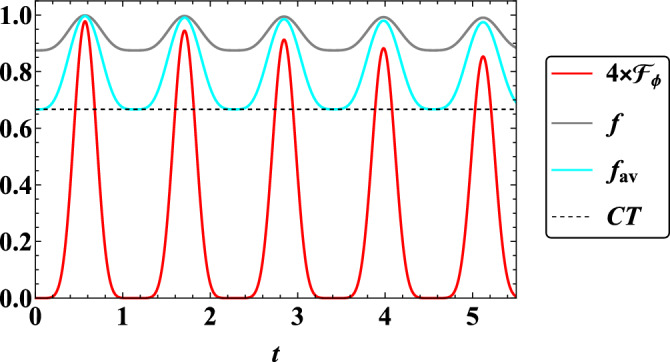
Dynamics of quantum Fisher information $\mathcal {F}_{\phi }$ with respect to $\phi$, fidelity *f*, and average fidelity $f_{av}$ when $B_1=0.5$, $\omega _0=1$ $G=1$, $x_j=1$, $J_x=2$, $J_y=0.1$, $\gamma =\lambda =0.001$, $\theta =\pi /6$, and $\phi =\pi$. CT (Black dotted line) represents classical threshold of teleportation.

**Figure 8:**
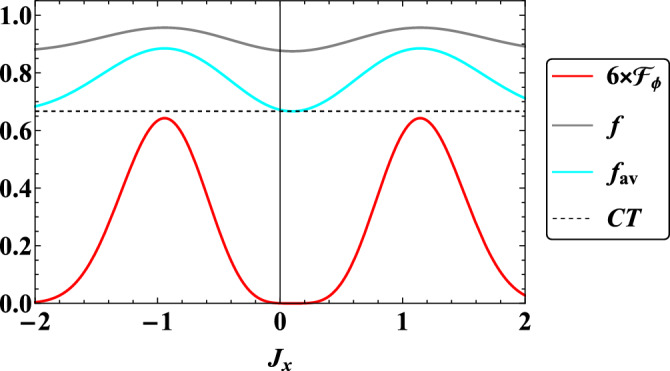
The qualitative behaviors of quantum Fisher information $\mathcal {F}_{\phi }$ with respect to $\phi$, fidelity *f*, and average fidelity $f_{av}$ in terms of $J_x$ when $B_1=0.5$, $\omega _0=0.8$ $G=1$, $x_j=1$, $J_y=0.1$, $\gamma =\lambda =0.001$, $\theta =\pi /6$, and $\phi =\pi$. In addition, we set $t=1$. CT (Black dotted line) represents classical threshold of teleportation.

In the last step, which is one of the most important achievements of this article, a comparison of the qualitative behaviors of quantum Fisher information $\mathcal {F}_{\phi }$ according to $\phi$, fidelity *f* and average fidelity $f_{av}$ in two-qubit quantum teleportation in the present model in terms of time *t* (Fig. 7) and in terms of the interaction coefficient $J_x$ (Fig. 8) is plotted. In both figures, it can be observed that the average fidelity $f_{av}$ and fidelity *f* are higher than the classical threshold *CT*, which indicates that quantum teleportation based on the present model is successful in the presence of a magnetic field gradient. Now we go to the subject of quantum sensing. In both figures, it can be seen that the qualitative behavior of phase quantum estimation is completely similar to the behavior of fidelity and average fidelity in such a way that their minimum and maximum points of qualitative behavior coincide with each other. Therefore, wherever we have the best quality of quantum teleportation, then the best information extraction from the phase of the initial state of quantum teleportation also occurs. In addition, the behaviors of all three above-mentioned criteria are oscillating, and this indicates that in some intervals when the behaviors have an increasing rate, the dynamics of the system will be non-Markovian, and at intervals when the behaviors have a decreasing rate, the dynamics of the system refer to Markovian. In intervals when the dynamic is Markovian, it means that information continuously flows from the system to the environment, while if there is a backflow of information from the environment to the system, this process has a non-Markovian dynamic, which is caused by the existence of quantum memory^56–58^. Since the non-Markovian dynamic of the system is determined from the information flow, witnesses based on QFI in^82^ and fidelity in^83^ have been suggested. According to them, a flow of QFI is defined as $I_{\phi }(t):= d\mathcal {F_{\phi }}/dt$, that if we have $I_{\phi }(t)>0$ for some interval *t*, then the time evolution is called non-Markovian. Furthermore, a flow of fidelity is introduced as $I_f(t):= df/dt$, that if we have $I_f(t)>0$ for some interval *t*, then the dynamic refers to non-Markovian. These flows of information respect to QFI and fidelity are valid for this work.

## Conclusion

Quantum teleportation enables the transmission of a desired quantum state between two locations, making it valuable for quantum communications. In this article, we investigated the quantum teleportation based on the Heisenberg XYZ chain with a magnetic-field gradient affected by intrinsic decoherence. An atomic spin chain is primarily coupled to the linear magnetic field gradient in the x-direction, with the assumption that the magnetic field varies linearly with the atom’s position.

Using the concepts of quantum threshold in quantum teleportation, fidelity and average fidelity in the current model were investigated and we were able to improve the quality of quantum teleportation and maintain fidelity and average fidelity in the quantum region by using various system variables.

In addition, we investigated remote quantum sensing according to the concept of remote quantum estimation with regard to the output state of two-qubit quantum teleportation. This in-depth investigation allowed us to clearly reveal the concepts of information flow that led to the clarification of the concept of non-Markovian time evolution of the system in the current model using evidence of non-Markovian dynamics such as fidelity and quantum Fisher information (QFI). In addition, we showed that in the presence of a magnetic field gradient and under the influence of intrinsic decoherence, an improved quantum teleportation can be obtained along with the extraction of improved information from the initial state phase of teleportation. It was found that in the intervals when the maximum amount of qualitative behaviors of fidelity and average fidelity occurs, the best information extraction from the initial state phase also occurs.

These results can be very useful in the implementation of experimental quantum teleportation^18,84,85^ and the selection of desired resources^23^. Moreover, the results of this paper can play valuable role in quantum remote sensing^86^.

## Data Availability

All data generated or analyzed during this study are included in this article.

## Abbreviations

KSEWA: Kaplan-Shekhtman-Entin-Wohlman-Aharony
DM: Dzyaloshinskii-Moriya
POVM: Positive operator valued measure
SLD: Symmetric logarithmic derivative
CT: Classical threshold
CR: Cramér-Rao
QFI: Quantum Fisher information
HSS: Hilbert-Schmidt speed
